# One-Pot Synthesis of UPy-Functionalized Nanocellulose under Mechanochemical Synergy for High-Performance Epoxy Nanocomposites

**DOI:** 10.3390/polym14122428

**Published:** 2022-06-15

**Authors:** Hanchen Wang, Jiayin Wu, Biao Huang, Qi-Lin Lu

**Affiliations:** 1 Fujian Key Laboratory of Novel Functional Textile Fibers and Materials, Minjiang University, Fuzhou 350108, China; wanghc08@163.com (H.W.); wujiayin@fafu.edu.cn (J.W.); 2 College of Material Engineering, Fujian Agriculture and Forestry University, Fuzhou 350002, China

**Keywords:** UPy-functionalized nanocellulose, one-pot method, mechanochemical synergy, molecular bridge, epoxy nanocomposites

## Abstract

The high strength, high specific surface area, excellent biocompatibility, and degradability of nanocellulose (NCC) make it a potential reinforcing phase for composite materials. However, the polyhydroxyl property of NCC renders it prone to self-aggregation and it has weak interfacial compatibility with non-polar substrates, limiting its enhancement performance for composite materials. Therefore, based on the high reactivity of NCC, the chemical modification of NCC to introduce functional groups is the basis for effectively reducing its self-aggregation, improving its interfacial compatibility with the polymer matrix, and creating nanocellulose-based functional materials. The existing functional modifications of NCC have limitations; they require cumbersome steps, generate low yields, and are environmentally unfriendly. Herein, ureido-pyrimidinone (UPy) was introduced to NCC through a sustainable and high-efficiency avenue formed by the mechanochemical synergy of microwaves and ultrasonication. The obtained UPy-modified nanocellulose (NCC-UPy) exhibited a rod-like shape, with a length of 200–300 nm and a width of 20–30 nm, which presented oriented and stable dispersion in an aqueous medium, and the zeta potential reached −40 mV. Moreover, NCC-UPy had good thermostability (>350 °C) and high crystallinity (82.5%) within the crystal type of cellulose I. Using the as-prepared NCC-UPy as a molecular bridge, it was organically combined with epoxy resin through multiple hydrogen bonds to construct a nanocomposite membrane with superior mechanical strength and thermal stability. The results revealed that NCC-UPy dispersed uniformly in the epoxy matrix without aggregating and that the interfacial compatibility was good, leading to an 87% increase in the tensile strength of the formed nanocomposite membrane when 0.5 wt.% NCC-UPy was loaded. It was proved that NCC-UPy had remarkable reinforcing potential and effective stress transfer capacity for composites. Consequently, this study may open the door to the development of a one-pot green approach for undertaking the functional modification of NCC, and it is of great significance for the development of NCC-based nanocomposites.

## 1. Introduction

Epoxy resin has been extensively applied in the fields of packaging, coating, electronic devices, and civil construction because of its excellent properties, including chemical corrosion resistance, adhesiveness, electrical insulation, and processing flexibility. Epoxy resin can be crosslinked and cured with various active hydrogen-containing compounds to form three-dimensional network structures due to the chemical activity of epoxy groups. Nevertheless, epoxy resin is fragile and has a poor anti-impact damage performance; brittle fractures occur easily in some extreme environments, limiting its possible applications. Targeting this limitation, the modification of epoxy resin, through the addition of nanoparticles that possess specific qualities that improve epoxy resin’s performance, is a current research hotspot.

As a natural polymer, nanocellulose (NCC) is widely used as reinforcing phase to enhance the mechanical properties of composite materials owing to its high strength, high specific surface area, excellent degradability, and biocompatibility [1,2,3]. NCC belongs to a class of biomass tissues that have a natural affinity for composite materials and can form a “self-adaptive structure” in composites that weakens local stress at the interface. Under the effects of local stress, NCC particles slip along the surface of filling materials to repair the fractured bonds and form new bonds, maintaining a certain adhesive strength between the polymer matrix and the filling material, thereby reducing damage to the composites. Consequently, NCC has been extensively used in polymers such as polyvinyl alcohol, starch, rubber, and epoxy resin to develop nanocomposites with specific properties [4,5]. Ruiz revealed that NCC could be combined with epoxy resin through hydrogen bonding between the surface hydroxyl groups of NCC and epoxy groups of epoxy resin to form a dense, three-dimensional network structure, greatly enhancing the mechanical and thermodynamic properties of epoxy resin [6]. However, NCC is prone to self-aggregation in the matrix due to the abundant hydroxyl groups, hindering the interfacial compatibility between NCC and the matrix and limiting its enhancement performance for composite materials. To alleviate this self-aggregation, the chemical modification of NCC as a means of introducing functional groups that reduce agglomeration and improve the interfacial compatibility between NCC and the matrix is essential. Jue performed a chemical modification of NCC by combining it with a silane coupling agent, following which the modified NCC was added into the epoxy resin; the results showed that the storage modulus and elastic modulus of the epoxy resin increased significantly [7]. Unfortunately, the existing functional modifications to NCC have limitations: they require cumbersome steps, generate low yields, and are environmentally unfriendly. Herein, ureido-pyrimidinone (UPy) was introduced to NCC through a sustainable and high-efficiency avenue formed by the mechanochemical synergy of microwaves and ultrasonication.

UPy is widely used in supramolecular polymers due to its strong bonding ability [8,9]. While UPy is often introduced to synthetic polymers to construct a functional supramolecule, the grafting of UPy with NCC to promote the oriented and stable dispersion of NCC in the development of nanocomposites has received limited attention. The combining of cellulose chemistry with supramolecular chemistry to obtain functionalized NCC and render it as a molecular bridge in the construction of epoxy nanocomposites with excellent performance qualities was investigated in this study. The one-pot reaction is a high-efficiency and simple method that can be used for the synthesis and functionalization of natural macromolecules. Inspired by the superior advantages of the one-pot synthesis strategy, UPy-modified NCC (NCC-UPy) was synthesized through microwave–ultrasonication synergy in this study. In the one-pot processing, the chemical action, mechanical force, and thermal effect created by microwave–ultrasonication combined to induce mechanochemical synergy, leading to a significant increase in the reaction efficiency and productivity of NCC-UPy. Moreover, the obtained NCC-UPy was used as a molecular bridge, combining with the epoxy matrix to form a supramolecular nanocomposite membrane based on the strong multiple hydrogen bonds between NCC-UPy and epoxy. The hydrogen bonding was formed between –OH and epoxy groups as well as -UPy groups, developing a supramolecular structure in the epoxy nanocomposites that dramatically enhanced the mechanical strength and flexibility of the epoxy resin. The morphology, structure, and mechanical and thermal performances of the epoxy nanocomposite membrane were also characterized in order to examine its formation mechanism and the reinforcing and toughening role of NCC-UPy in the epoxy matrix.

## 2. Experimental Model

### 2.1. Materials

Bisphenol A epoxy resin was provided by Dow Chemical Pacific Singapore, Singapore. 2-amino-4-hydroxyl-6-methylpyrimidine, n-pentane, dibutyltin dilaurate (DBTDL), hexamethylene diisocyanate, diethylenetriamine (DETA, C_4_H_13_N_3_), acetone, and dimethylformamide (DMF) were purchased from Sinopharm Chemical Reagent Co., Ltd. (Shanghai, China). All chemicals used were of analytical grade, without any further purification.

### 2.2. Preparation of NCC-UPy

**Figure 1:**
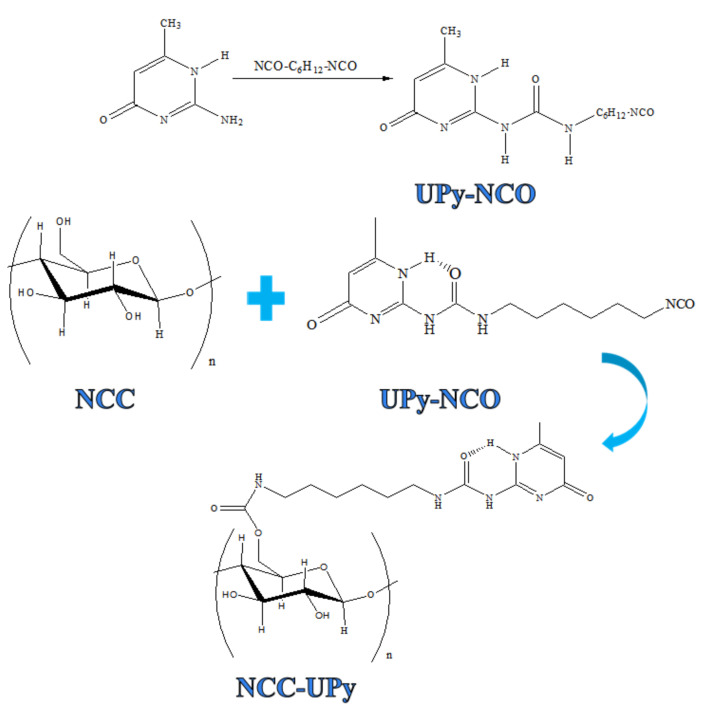
Schematic illustration of the UPy-functionalized NCC.

First, 0.1 mol 2-amino-4-hydroyl-6-methylpyrimidine and 0.6 mol diisocyanate solution were added into a microwave–ultrasound instrument and then preformed at 100 °C for 16 h under a microwave power of 600 W and an ultrasonic power of 700 W to obtain UPy-NCO. The resultant UPy-NCO solution was purified and filtered with n-pentane to remove reagents and collect the precipitate; subsequently, it was dried in a vacuum oven at 50 °C to collect the white powder, namely, UPy-NCO. NCC was prepared using our previously described mechanochemical method [10]. Then, 1 g NCC, 2 g UPy-NCO, and 0.05 g DBTDL were added into the DMF, and then the mixture was preformed at 60 °C for 14 h under a microwave power of 600 W and an ultrasonic power of 700 W. The resultant suspension was purified through centrifugation with deionized water at 10,000 rpm for 10 min in order to remove the DBTDL and the DMF and collect the NCC-UPy suspension (Figure 1).

### 2.3. Construction of Epoxy Nanocomposite Membrane

**Figure 2:**
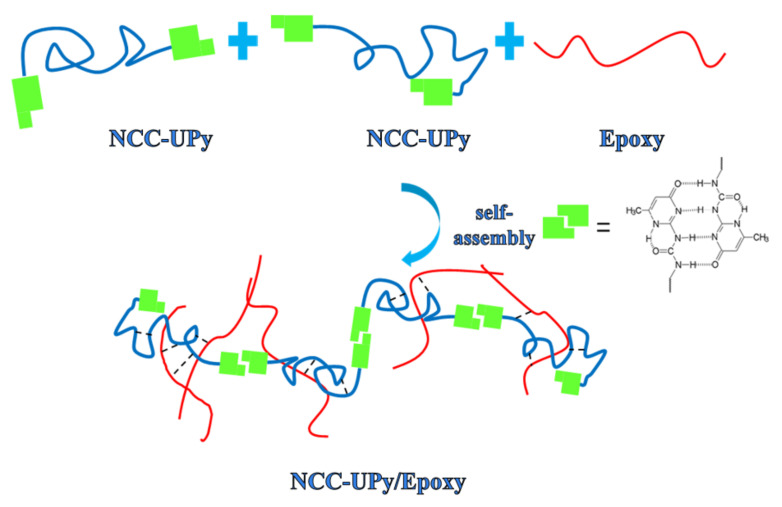
Schematic illustration of the formation of an epoxy nanocomposite membrane based on NCC-UPy and epoxy.

We added 1 g NCC-UPy into the acetone and sonicated it for 30 min to disperse it evenly in the acetone. The epoxy resin was diluted with acetone (15 wt.%) to increase its fluidity. Then, 20 g epoxy resin and 10 g DETA were added into the acetone solvent containing NCC-UPy and sonicated at 50 °C for 2 h to form a uniform suspension. After removing the bubbles, the mixture was poured into PTFE Petri dishes with a diameter of 10 cm for curing at room temperature for 48 h to form the NCC-UPy/epoxy nanocomposite membrane. For comparison purposes, the epoxy membrane was prepared under the same conditions in the absence of NCC-UPy. Moreover, to explore the effect of NCC-UPy on the properties of the epoxy nanocomposite membrane, under the same conditions, NCC-UPy/epoxy nanocomposite membranes with different amounts of NCC-UPy (0.1, 0.2, 0.3, 1 wt.%) added to the epoxy resin were prepared (Figure 2).

### 2.4. Performance Characterization

The morphology of NCC-UPy was observed using FESEM (SU8010, Hitachi, Ltd., Tokyo, Japan) and a transmission electron microscope (Hitachi-H7650, Hitachi, Ltd., Tokyo, Japan). The chemical structure of NCC-UPy was analyzed using FT-IR and NMR. FT-IR spectra were recorded using the KBr disk method via a Nicolet 380 FT-IR spectrometer (Thermo Electron Instruments Co., Ltd., Madison, WI, USA). Solid-state ^13^C NMR spectra were acquired on a Bruker Avance III 500 spectrometer (Bruker Biospin AG, Fallanden, Switzerland). A UV–Vis spectrophotometer was used to determine the degree of substitution of NCC-UPy. An elemental analyzer (Vario MICRO, Elementar Analysensysteme GmbH, Langenselbold, Germany) was used to detect the levels of carbon, hydrogen, oxygen, and nitrogen elements in NCC-UPy. The crystalline structures of NCC and NCC-UPy were investigated using X-ray diffraction (XRD) analysis on an X-ray diffractometer (Philips-FEI, FEI Company, Hillsboro, OR, USA) with Cu Kα radiation. Diffractograms were collected in the range of 2θ = 6–90°at a scanning rate of 0.1°s^−1^. The zeta potential values were tested using a Zetasizer (NETZSCH-STA449F3, NETZSCH, Munich, Germany) instrument. The thermodynamic properties of the composite membrane were investigated using a STA449F3 thermal analyzer (NETZSCH, Munich, Germany). A N_2_ atmosphere was used, the flow rate was 30 mL/min, the temperature range was 25–600 °C, and the heating rate was 10 °C/min. The morphology of the epoxy nanocomposites was observed using FESEM (SU8010, Hitachi, Ltd., Tokyo, Japan) at an accelerating voltage of 1.0 kV. The nanocomposites were fractured in liquid nitrogen to expose their cross-sections and then sputtered with gold before observation. The mechanical properties of NCC-UPy/epoxy nanocomposite membrane were examined using a universal testing machine (Instron 5567, Norwood, MA, USA) at room temperature. Dynamic mechanical analysis (DMA) of the NNC-UPy/epoxy nanocomposite membrane was conducted on a Q 800 DMA (TA, New Castle, DE, USA) from 20 to 150 °C.

## 3. Results and Discussion

### 3.1. Morphology and Dispersion Stability

**Figure 3:**
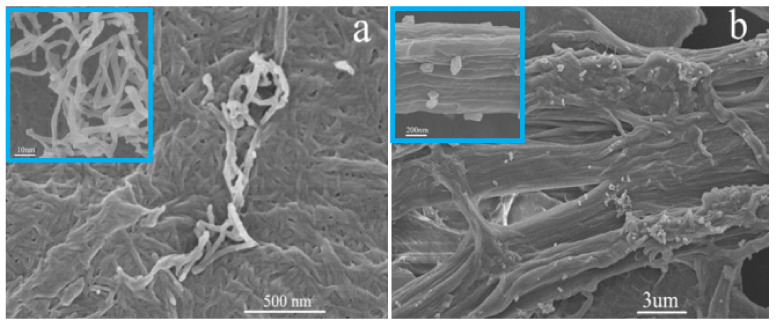
(**a**) SEM micrograph of NCC; (**b**) SEM image of NCC-UPy.

SEM images were used to investigate the changes in morphology and microstructure of NCC and NCC-UPy. As is depicted in Figure 3a,b, NCC presented as rod-like shapes with a diameter of about fifty nanometers and a length of several hundred nanometers. Obvious agglomeration occurred during the drying process; this is likely due to the large number of hydroxyl groups on the surface of NCC [11]. NCC-UPy presented as a long rod shape with a rough fiber surface, a significant increase in size, and a large aspect ratio. Moreover, NCC-UPy tended to bundle into rod-like nanocrystals and was mostly aligned in the form of aggregates, which could be attributed to the highly oriented multiple hydrogen bonds formed through the -UPy groups of NCC-UPy. The orienting ordered arrangement of NCC-UPy may have been the result of hydrogen bonding between the modified nanocrystals that held the nanocellulose rods together [12,13]. The oriented morphology enabled NCC-UPy to provide better reinforcement for composites.

**Figure 4:**
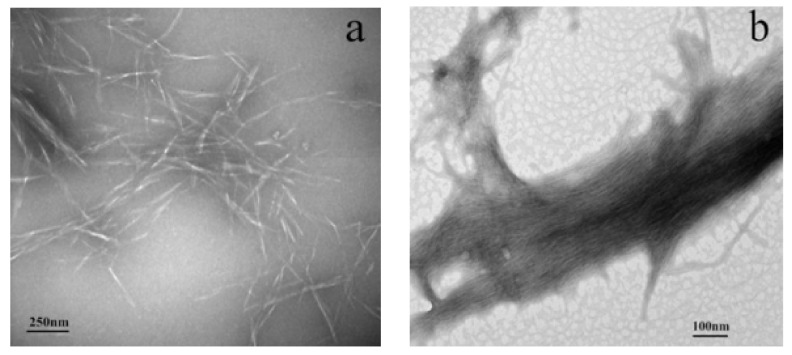
TEM micrograph of NCC (**a**) and NCC-UPy (**b**).

The morphology of the NCC and UPy-NCC suspensions could be observed more clearly through TEM images. As can be seen in Figure 4a,b, the short, rod-like NCC had a diameter of 25–50 nm and a length of 200–300 nm. Self-aggregation occurred, and parts of NCC aggregated to form a network structure due to the hydrogen bonding between hydroxyl groups. After the modification of -UPy, the obtained NCC-UPy formed long rod shapes, and the NCC-UPy nanofibers gathered together in a directional arrangement to form directional aggregates. This was because of the relatively facile formation of strong quadruple hydrogen bonds between the -UPy groups of NCC-UPy. The high orientation of hydrogen bonds induced the orienting ordered arrangement of NCC-UPy.

In general, NCC has a negative value of zeta potential since it contains hydroxyl and uronic acid groups. According to the zeta potential measurements, the zeta potential of NCC was found to be −16.8 ± 0.5 mV; interestingly, after modification with -UPy, the zeta potential of NCC-UPy reduced to −40 ± 0.3 mV. The decrease in zeta potential values clearly confirmed that the electrostatic repulsion between NCC-UPy nanoparticles was enhanced, the hydrogen bonding was weakened, and NCC-UPy had good dispersibility [14]. Consequently, the superior dispersion stability of NCC-UPy was beneficial for the prevention of agglomeration in the epoxy matrix [15].

### 3.2. FTIR Analysis

**Figure 5:**
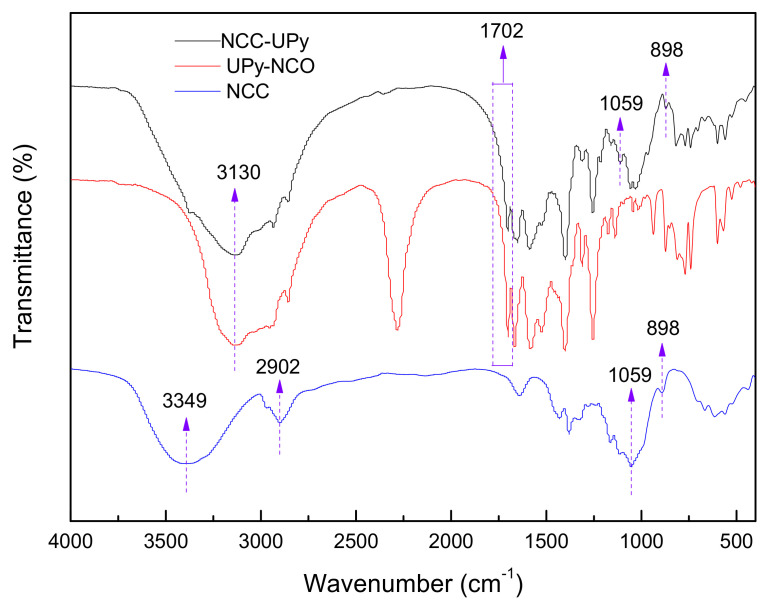
FTIR spectra of NCC and NCC-UPy.

The types of functional groups of NCC, UPy-NCO and NCC-UPy were evaluated using FTIR; the results are shown in Figure 5. The wide absorption band at around 3349 cm^−1^ belonged to the O–H stretch which was affected by hydrogen bonds. The typical characteristic peaks at 1160 and 1110 cm^−1^ corresponded to the stretching vibration of C–C in the cellulose and the stretching vibration of the glucose ring, respectively [16,17]. The absorption band at 898 cm^−1^ was associated with vibration absorption of heterohead carbon (C1) [18], which was generated by β-glycosidic bonds between anhydroglucose units in cellulose. These characteristic peaks of cellulose were contained in the FTIR spectrum of NCC-UPy, showing that NCC-UPy still had the basic structure of cellulose. Interestingly, UPy-NCO had a strong absorption peak at 2250 cm^−1^, which corresponded to the characteristic peak of the –NCO group [19]. While the peak disappeared in the spectrum of NCC-UPy, a new absorption peak appeared at 1702 cm^−1^, corresponding to the absorption peak of the carbonyl group (–C=O) in the –OOCNH– group [20,21]. Such a phenomenon indicated that UPy-NCO had a chemical reaction with –OH in nanocellulose to form a urethane bond (–OOCNH–). Other bands at 2860 and 2951 cm^−1^ were attributed to the absorption peak of -urea carbonyl (–NOCN–) in the UPy group and the N–H symmetric stretching vibration of the imino group in the –OOCNH– group, respectively [22]. The results of FTIR spectra conclude that the -UPy groups were successfully grafted onto NCC.

### 3.3. Solid-State ^13^C NMR Analysis

**Figure 6:**
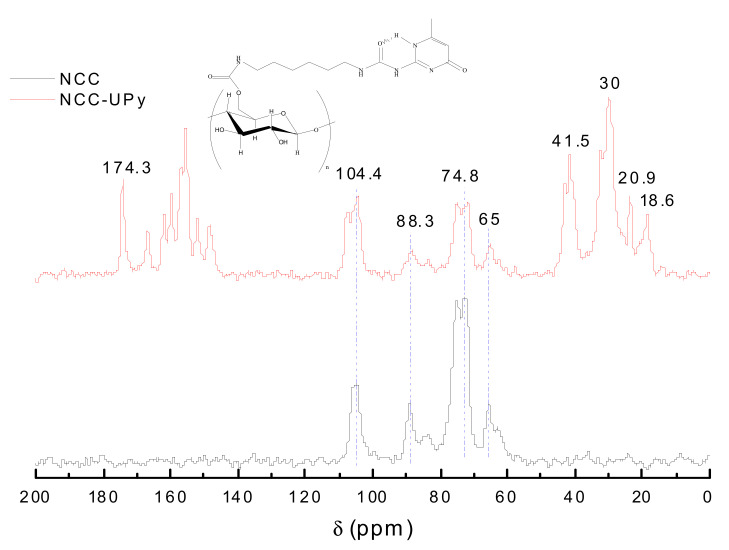
^13^C NMR spectra of NCC and NCC-UPy.

Solid-state ^13^C NMR spectroscopy was also used to determine the structural differences between NCC and NCC-UPy. As is shown in Figure 6, the characteristic absorption signals of NCC appeared at 104.4, 88.3, 74.8, and 65 ppm, which corresponded to the resonance absorption signals of C1, C4, C2, C3, C5, and C6 of cellulose. The C6 (62.6 ppm) and C4 (83.5 ppm) signals were assigned to carbons of glucopyranose rings in noncrystalline regions [23]. The strong absorption peaks at δ = 69–80 ppm belonged to the C2, C3, and C5 cyclic carbons not connected to glycosidic bonds. On the other hand, the characteristic absorption signals of NCC could be detected in the NCC-UPy spectrum, indicating that, after being modified by the UPy, NCC-UPy still retained the basic skeleton of cellulose. NCC-UPy had new resonance absorption signals at 174.3 ppm and 15–45 (18.6, 20.9, 30, 41.5) ppm, which were assigned to the chemical shifts of carbonyl groups and methylene groups of the -UPy molecular chain, respectively. Moreover, there was an obvious loss of resolution at 88.3 and 65 ppm, implying that a covalent bond was formed between NCC and -UPy [24]; i.e., the -UPy group was successfully grafted to the surface of NCC.

### 3.4. Degree of Substitution Measurement

**Figure 7:**
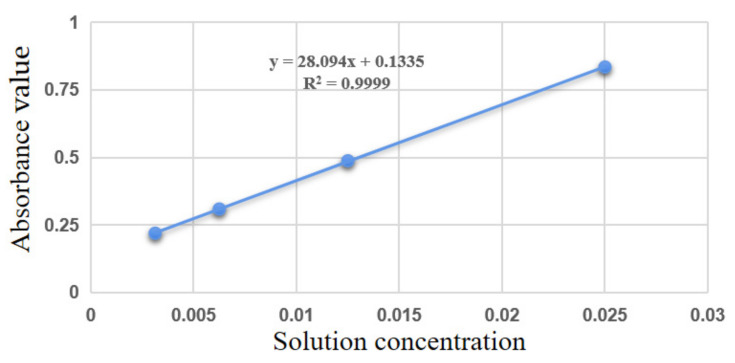
Absorbance curves of NCC-UPy at 282 nm as a function of concentration.

The curve of absorbance versus concentration of the NCC-UPy solution at a wavelength of 282 nm is shown in Figure 7, from which the corresponding equation between absorbance and concentration was obtained, as shown in Equation (1):

(1) y=28.094x+0.1335

where *y* is the absorbance value of the NCC-UPy solution at 282 nm, and *x* is the solution concentration of the NCC-UPy.

Through the UV test, it could be observed that the NCC-UPy solution had a maximum absorption peak at the wavelength of 282 nm, which disappeared in the NCC solution of the same concentration, indicating that 282 nm was the UV absorption wavelength of the -UPy group. The absorbances at 282 nm of NCC-UPy, UPy-NCO, and NCC with a concentration of 0.125 mg/mL were measured, and then the degree of substitution of the -UPy group in NCC-UPy (17.38%) was calculated. The elemental analysis results of NCC and NCC-UPy are shown in Table 1. While the nanocellulose did not contain any N element before modification, the content of N element within the obtained NCC-UPy molecule reached 13.96%. Due to the substitution of hydroxyl groups on the surface of nanocellulose and the long carbon chain of the -NCO group during the chemical modification, the content of C and H elements in the modified nanocellulose increased to 47.89% and 7.59%, respectively, while the oxygen content dropped to 30.56%. As confirmed by the above results, the -UPy group was successfully grafted onto the nanocellulose.

### 3.5. Crystal Structure

**Figure 8:**
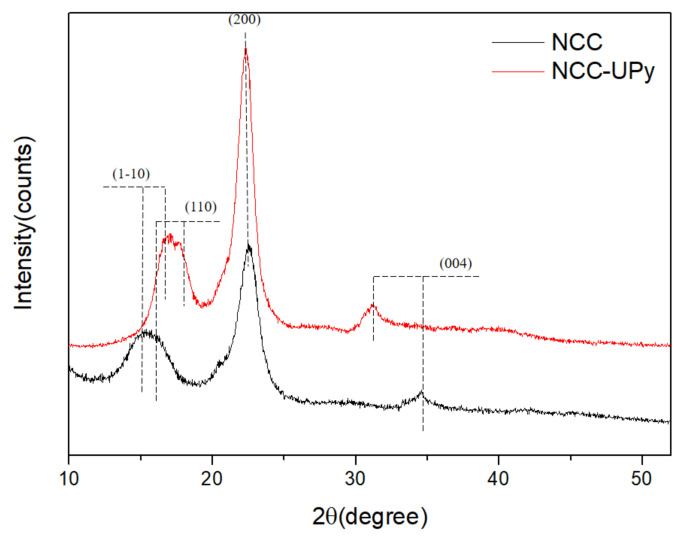
XRD spectra of NCC and NCC-UPy.

The XRD patterns of NCC and NCC-UPy are shown in Figure 8. All samples exhibited four diffraction peaks near 2θ = 15°, 16.5°, 22.7°, and 35°, corresponding to (1–10), (110), (200), and (400) diffraction planes of cellulose lattice, respectively, which suggests that the crystalline type of NCC-UPy was not altered in the manufacturing process and that NCC-UPy retained the cellulose I crystal form [25]. The calculated crystallinity indexes of NCC and NCC-UPy were 71.6% and 82.5%, respectively, demonstrating the hydrolysis of amorphous parts under the microwave–ultrasonication synergy. The preservation of the crystalline nature of NCC, made possible through this process, would be beneficial for many structural applications of NCC-UPy. The high crystallinity of NCC-UPy is expected to be significant for the enhancement of nanocomposites’ mechanical performance since higher crystallinity is associated with higher tensile strength [26].

### 3.6. Thermostability of NCC-UPy

**Figure 9:**
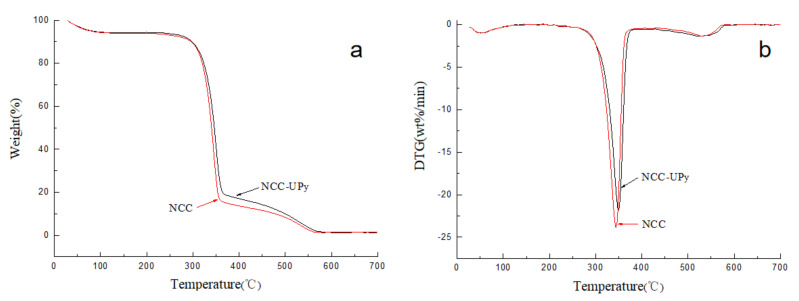
(**a**) TG and (**b**) DTG curves of NCC and NCC-UPy.

As is shown in Figure 9a, the weight loss of 6 wt.% below 120 °C for NCC and NCC-UPy could be attributed to water evaporation, the main thermal decomposition temperature of the molecular structure ranged from 280 °C to 380 °C, and carbonation occurred when the temperature rose above 400 °C. The calculated onset decomposition temperatures of NCC and NCC-UPy were 322.4 °C and 326.6 °C, respectively, and the maximum decomposition temperatures were 343.6 °C and 350.2 °C, respectively (Figure 9b). Moreover, as shown in Table 2, the char residue of NCC-UPy at 700 °C was 1.52 wt.%, which is higher than that of NCC (1.32 wt.%). The TG and DTG results mean that the thermal stability of NCC-UPy was slightly increased compared with that of NCC, which derives from the enhanced crystallinity of NCC-UPy [27]. The excellent thermostability of NCC-UPy means it has high developmental potential in nanocomposite applications.

### 3.7. Morphology of Epoxy Nanocomposites

**Figure 10:**
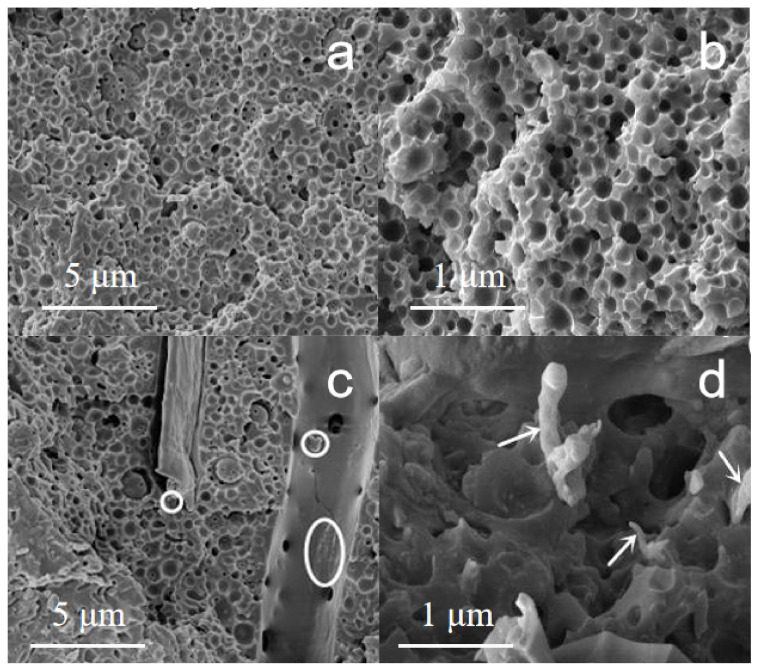
SEM images of the fractured surfaces of (**a**,**b**) neat epoxy membrane and (**c**,**d**) NCC-UPy/epoxy nanocomposite membrane.

Compared with the fractured surface of the neat epoxy membrane (Figure 10a,b), the NCC-UPy/epoxy nanocomposite membrane showed a rather rough, fractured surface, and more cracks were present (Figure 10c,d), indicating that the nanocomposite membrane had better toughness. The main reason for this was that when the nanocomposite membrane was subjected to external force, the uniformly dispersed NCC-UPy experienced crazing under the external force; the crazing extended sharply and branched rapidly after reaching a critical length to disperse the front-end stress of the single crazing crack. Therefore, the forward-extension crazing expanded to the surroundings, and, then, the epoxy matrix between the NCC-UPy components exhibited plastic deformation. In addition, parts of NCC-UPy fell off the epoxy matrix, consuming energy in the system and leading to a rougher fractured surface. Figure 10c shows that the cavities (small white circles) in the NCC-UPy/epoxy nanocomposite membrane were formed through the microphase separation of NCC-UPy and the epoxy matrix during the curing process, which promoted matrix shear deformation to produce shear zones and shear yield, thus consuming more energy and improving the mechanical strength. Figure 10d shows that NCC-UPy was wrapped in the epoxy matrix and could still firmly attach to the matrix even when the nanocomposite membrane was stretched and broken, suggesting that NCC-UPy had strong interfacial adhesion with the epoxy matrix, probably through the hydrogen bonding between –OH and epoxy groups [28]. Additionally, the aspect ratio of NCC-UPy was larger than that of NCC, which may have formed a mechanical interlocking effect to hinder the pull-out of NCC-UPy. The morphology analysis confirmed that NCC-UPy could restrict the free movement of epoxy resin and had good reinforcing and toughening effects on the epoxy matrix.

### 3.8. Mechanical Properties of Epoxy Nanocomposites

**Figure 11:**
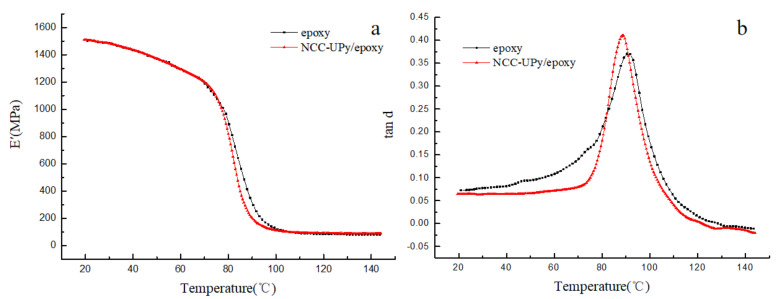
Storage modulus (**a**) and loss factor (**b**) as a function of temperature for epoxy nanocomposites.

Figure 11a,b shows the storage modulus and loss factor of NCC-UPy/epoxy as a function of temperature, respectively. Both NCC-UPy/epoxy and epoxy resin demonstrated the unique amorphous polymer characteristic, but NCC-UPy/epoxy had a slightly improved performance due to the rigid nature of NCC-UPy [29]. The storage modulus of NCC-UPy (1510 MPa) had a slight increase compared to that of epoxy resin (1500 MPa) at 90 °C. A possible reason for this was that the rigid NCC-UPy was finely embedded in the epoxy matrix and formed a stiff and continuous network by the hydrogen-bonded NCC-UPy and epoxy resin, with NCC-UPy particles acting as gluing agents between the epoxy particles [30]. Figure 11b shows that the glass transition temperatures (Tg) of NCC-UPy/epoxy and epoxy resin were 91 °C and 88 °C, respectively. The slight rise in Tg for NCC-UPy/epoxy could be attributed to the uniform dispersion of NCC-UPy in the epoxy matrix. This dispersion in the epoxy matrix induced an increase in crosslinking density [31]. The result revealed that the toughness and storage modulus of the NCC-UPy/epoxy nanocomposite membrane were enhanced with the addition of NCC-UPy.

**Figure 12:**
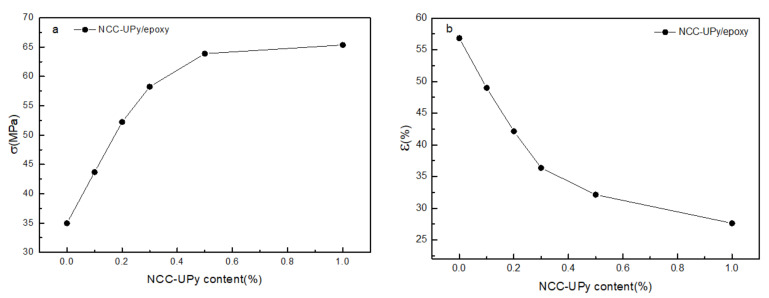
(**a**) Tensile strength and (**b**) elongation at break curves of NCC-UPy/epoxy composite films as a function of the cellulose content.

Figure 12a,b present the curves of the tensile strength and elongation at the break of the NCC-UPy/epoxy composite films as a function of NCC-UPy content, respectively. With increasing NCC-UPy content, the tensile strength of the NCC-UPy/epoxy membrane increased gradually while the elongation at the break decreased due to the large aspect ratio and high crystallinity of NCC-UPy. When the NCC-UPy content increased to 0.5 wt.%, the tensile strength of the NCC-UPy/epoxy membrane increased from 34.97 MPa of epoxy resin to 65.35 MPa, and the value increased by 86.87%. However, the elongation at the break decreased from 56.8% to 27.6%. Although previous studies documented the enhancing effects of modified NCC on epoxy, the effects were unsatisfactory when the modified NCC content was 0.5 wt.% (45.22 MPa) [28]. Other studies had to work at very high modified-NCC concentrations (about 50 wt.%) to obtain similar enhancements to those of NCC-UPy at this low concentration [32,33]. This was mainly attributed to the good dispersibility of NCC-UPy in the epoxy matrix as well as the strong interfacial bonding force between NCC-UPy and the epoxy induced by their multiple hydrogen bonds, meaning that part of the load applied to the epoxy was transferred to the high-strength NCC-UPy molecular chains. The hydroxyl, carbonyl, and imino groups of NCC-UPy could form O–H, C=O and N–H hydrogen bonds with the hydroxyl groups of epoxy molecular chains. As a result, the intramolecular and intermolecular hydrogen bonds of the epoxy molecular chains were weakened, and the density of mutual bonding and entanglement between epoxy molecular chains was reduced, leading to an improvement in the dispersibility of NCC-UPy in the epoxy matrix. On the other hand, the strong quadruple hydrogen bonds between -UPy units promoted the formation of physical crosslinking in the supramolecular nanocomposite membrane, and the free movement of epoxy at the cracks was limited during the stressing process; thus, the strength of NCC-UPy/epoxy was enhanced. However, the strong, rigid structure of NCC-UPy restricted the mobility of epoxy molecular chains, meaning that the elongation at break of the supramolecular nanocomposite membrane was reduced. In summary, the strain–stress analyses indicated that 0.5 wt.% NCC could significantly enhance the tensile strength of epoxy films without severely compromising the elongation at break of the supramolecular nanocomposite membrane. 

## 4. Conclusions

To realize comprehensive utilization of NCC and simple integration into nanocomposite manufacturing, NCC-UPy was produced via the mechanochemical synergy of microwaves and ultrasonication, reducing the necessity of lengthy reaction times and realizing a high-efficiency process. The obtained NCC-UPy presented an orienting ordered arrangement with superior dispersibility, good thermostability, and high crystallinity, beneficial attributes in its utilization in the construction of high-performance supramolecular nanocomposites. The NCC-UPy/epoxy nanocomposite membrane’s high mechanical performance was developed through the multiple hydrogen bonds between NCC-UPy and epoxy. It was proved that NCC-UPy played a role in molecular bridging and had a reinforcing effect on the mechanical capacity of epoxy resin, leading to a significant increase in tensile strength, even at low NCC-UPy loading. This simple and sustainable production method for functionalized nanocellulose, as well as the unique molecular bridging and reinforcing capacities of NCC-UPy, renders it a versatile nanomaterial. Therefore, the study may provide a new perspective on the directional design of nanocellulose and the selective construction of nanocellulose functional materials.

## Figures and Tables

**Table 1:** Elemental analysis results for NCC and NCC-UPy.

Sample	C%	H%	N%	O%
NCC	41.29	6.88	0	51.83
NCC-UPy	47.89	7.59	13.96	30.56

**Table 2:** Analysis results from TG and DTG curves.

Sample	Onset Temperature (°C)	Maximum Temperature (°C)	Char Residue (wt.%)
NCC-UPy	326.6	350.2	1.52
NCC	322.4	343.6	1.32

## Data Availability

The data presented in this study are available on request from the corresponding author.
